# Screening and characterization of the scFv for chimeric antigen receptor T cells targeting CEA-positive carcinoma

**DOI:** 10.3389/fimmu.2023.1182409

**Published:** 2023-05-25

**Authors:** Chengcheng Zhang, Linling Wang, Qianzhen Zhang, Junjie Shen, Xia Huang, Meiling Wang, Yi Huang, Jun Chen, Yanmin Xu, Wenxu Zhao, Yanan Qi, Yunyan Li, Yanjiao Ou, Zhi Yang, Cheng Qian

**Affiliations:** ^1^ Department of Hepatobiliary Surgery, Southwest Hospital, Army Medical University, Chongqing, China; ^2^ Chongqing Key Laboratory of Gene and Cell Therapy, Institute of Precision Medicine and Biotechnology, Chongqing Precision Biotech Co. Ltd., Chongqing, China

**Keywords:** chimeric antigen receptor T cells, carcinoembryonic antigen, single-chain fragment variable, affinity, cell therapy

## Abstract

**Introduction:**

Chimeric antigen receptor T (CAR-T) cell therapy presents a promising treatment option for various cancers, including solid tumors. Carcinoembryonic antigen (CEA) is an attractive target due to its high expression in many tumors, particularly gastrointestinal cancers, while limited expression in normal adult tissues. In our previous clinical study, we reported a 70% disease control rate with no severe side effects using a humanized CEA-targeting CAR-T cell. However, the selection of the appropriate single-chain variable fragment (scFv) significantly affects the therapeutic efficacy of CAR-T cells by defining their specific behavior towards the target antigen. Therefore, this study aimed to identify the optimal scFv and investigate its biological functions to further optimize the therapeutic potential of CAR-T cells targeting CEA-positive carcinoma.

**Methods:**

We screened four reported humanized or fully human anti-CEA antibodies (M5A, hMN-14, BW431/26, and C2-45), and inserted them into a 3rd-generation CAR structure. We purified the scFvs and measured the affinity. We monitored CAR-T cell phenotype and scFv binding stability to CEA antigen through flow cytometry. We performed repeated CEA antigen stimulation assays to compare the proliferation potential and response of the four CAR-T cells, then further evaluated the anti-tumor efficacy of CAR-T cells ex vivo and in vivo.

**Results:**

M5A and hMN-14 CARs displayed higher affinity and more stable CEA binding ability than BW431/26 and C2-45 CARs. During CAR-T cell production culture, hMN-14 CAR-T cells exhibit a larger proportion of memory-like T cells, while M5A CAR-T cells showed a more differentiated phenotype, suggesting a greater tonic signal of M5A scFv. M5A, hMN-14, and BW431/26 CAR-T cells exhibited effective tumor cell lysis and IFN-γ release when cocultured with CEA-positive tumor cells *in vitro*, correlating with the abundance of CEA expression in target cells. While C2-45 resulted in almost no tumor lysis or IFN-γ release. In a repeat CEA antigen stimulation assay, M5A showed the best cell proliferation and cytokine secretion levels. In a mouse xenograft model, M5A CAR-T cells displayed better antitumor efficacy without preconditioning.

**Discussion:**

Our findings suggest that scFvs derived from different antibodies have distinctive characteristics, and stable expression and appropriate affinity are critical for robust antitumor efficacy. This study highlights the importance of selecting an optimal scFv in CAR-T cell design for effective CEA-targeted therapy. The identified optimal scFv, M5A, could be potentially applied in future clinical trials of CAR-T cell therapy targeting CEA-positive carcinoma.

## Introduction

Chimeric antigen receptor T lymphocyte (CAR-T) therapy has shown encouraging and convincing antitumor effects with a high complete remission (CR) rate in refractory and relapsed hematological malignancies, especially leukemia (1, 2). Breakthroughs are also being attempted in solid tumors (3–5). Carcinoembryonic antigen (CEA) is an early tumor-specific marker for human colon cancer (6). Subsequent studies have shown that CEA is expressed in several types of solid tumors, such as colorectal, gastric, lung, breast, pancreatic, and ovarian carcinomas (7). CEA serves as a tumor marker for screening, diagnosis, and prognosis prediction in many cancers (8–13). Its safety and feasibility as a target for chimeric antigen receptor T (CAR-T) cell therapy have been demonstrated in multiple preclinical and clinical trials for the treatment of CEA-positive solid tumors (14–20).

The potential for off-target and on-target/off-tumor side effects are major concerns of CAR-T therapy (21, 22). Although CEA, as a tumor-associated antigen, is highly expressed in malignant tumors, it is also physiologically expressed at low levels in some normal tissues, such as the tongue epithelium, tracheal mucosa, and gastrointestinal tract (23). Thus, the influence of CAR-T cells on these normal tissues must be considered. Amino acid mutations of monoclonal antibodies have generated a series of antibodies with different affinities but targeting the same epitope (24). Among these antibodies, scFvs with decreased affinity were found to exhibit potent antitumor efficacy and better safety in related CAR-T cells (25, 26). However, scFvs derived from different hybridomas targeting diverse epitopes have produced conflicting results regarding CAR-T cell therapy. High-affinity scFvs resulted in better tumor eradication (27, 28). Thus, the selection of appropriate epitopes and affinity for CEA-targeted CAR-T cells is crucial in reducing adverse reactions while improving efficacy.

Murine scFvs are likely to induce humoral (e.g., human anti-mouse antibodies, HAMA) and cellular anti-CAR immune responses, leading to lethal events such as anaphylaxis and attenuated CAR-T efficacy. (29, 30). The humanization of scFvs significantly reduced the immunogenicity and increased the persistence and safety of CAR-T cells compared to those of murine CAR-T cells (31). Humanized CAR-T cells have shown superior clinical therapeutic efficacy (32) and sustained antitumor ability in patients who relapsed after murine CAR-T cell treatment (33, 34). Therefore, humanization is a critical factor in scFv selection for CAR-T cell therapy.

T-cell exhaustion attenuates the efficacy of CAR-T cells and shortens their persistence *in vivo*. Several scFvs even exhibit early exhaustion due to scFv clustering, CAR-dependent tonic signal, and antigen-independent signal transduction (35). It has been reported that CARs with different scFvs have different cell phenotypes and functions (36). Therefore, screening for scFvs with low self-activation and sustained function is necessary to prevent T-cell exhaustion and ensure optimal CAR-T cell therapy.

In this study, we selected four humanized monoclonal antibodies (M5A, hMN-14, C2-45, and BW431/26) based on the binding domain position, humanization status, serum CEA blockade efficacy, reported affinity, and application frequency. We inserted the VH and VL domains of these four mAbs into a 3rd-generation CAR backbone (scFv-G4h-28TM-28BBZ) to evaluate and compare the phenotype, CAR expression level, proliferation, and function of each CAR-T cell type. Additionally, we measured the affinity of each scFv and performed assays to assess cytolysis targeting CEA-positive cells *in vitro* and potent control of tumors *in vivo*. Our findings highlight the distinct features and functions of each scFv targeting CEA, providing improved strategies to enhance the clinical efficacy of CEA-targeted CAR-T therapy.

## Materials and methods

### scFv purification and affinity measurement

**Figure 1:**
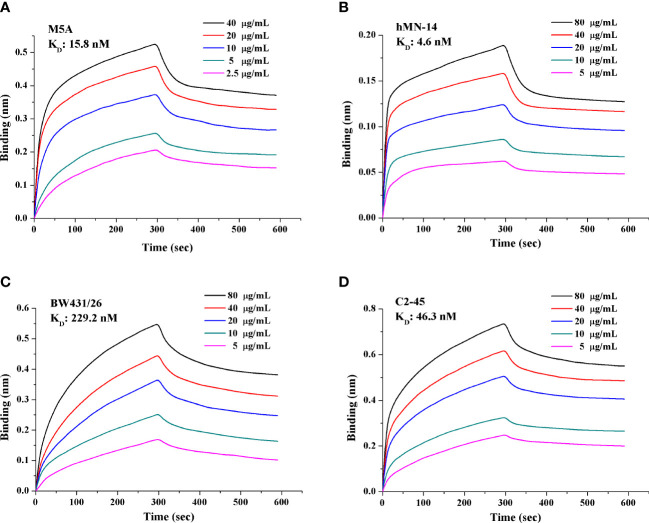
Affinity and binding kinetics of each scFv targeting CEA. The K_D_ of each scFv was obtained and calculated based on a gradient of 5 concentrations. **(A)** M5A, **(B)** hMN-14, **(C)** BW431/26, and **(D)** C2-45. A lower K_D_ value indicates higher affinity. Each experiment was conducted three independent times (n=3).

The His-tagged sequence of each of the 4 scFvs was inserted into a plasmid and transfected into HEK293 cells. The supernatant was collected after 6 days. Purification was conducted through filtration (0.22 μm) and absorption (nickel column). The purity of the purified protein was greater than 95%. The concentration was measured by the Bradford method. scFv affinity was measured using a FortéBio Octet Red 96 biolayer interferometry system (Pall, CA, U.S.). The detailed protocol followed the published literature (37). Anti-human IgG Fc (AHC) biosensors (Pall FortéBio, 1512121) were purchased and applied for measurement. Fc-tagged recombinant CEA (15 μg/ml; CEA-Fc, 11077-H03H-50, Sino Biological Inc.) was precoated on the biosensor. scFv was diluted twice in a gradient of 5 concentrations, with phosphate-buffered saline (PBS) as the negative control. Binding kinetics were calculated using FortéBio Data Analysis software (version 7.1) for y-axis alignment, reference subtraction, interstep correction, and Savitzky−Golay filtering. The association (kon, 1/Ms) and dissociation (koff, 1/s) rate constants were determined by fitting the association and dissociation data to a 1:1 model. The binding affinity (K_D_, nM) was calculated as koff/kon (**Figure 1**). The experiment was conducted three independent times.

### Tumor cell lines and CEA-expressing HEK293T cells

Cell lines with high (LS174T and LoVo), moderate (HT-29 and Caco-2), low (MCF7), and negative (RKO and SW620) CEA expressions were purchased from ATCC and cultured according to the corresponding protocols. STR identification of the main cell lines was carried out. CEA expression in each cell line was confirmed before the experiment. The pCMV-CEACAM5-GFP plasmid was obtained from Sino Biological Inc. (HG11077-ACG) and transfected into HEK293T cells to obtain cells with different CEA expression levels. Then, 6 μg, 3 μg, and 1.5 μg of the plasmid were transfected into HEK293T cells to produce high, medium, and low CEA expression, respectively. CEA expression in these HEK293T cells was confirmed by FACS, and the cells were used for cytolytic assays. Untransfected HEK293T cells were used as a control.

### Generation of lentivirus and CAR-T cells

**Figure 2:**
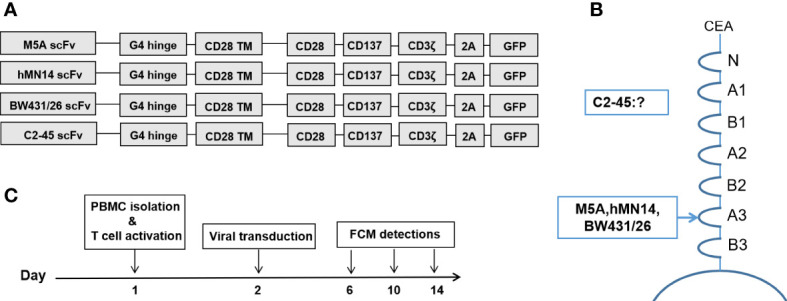
CAR structures and detection schedule during CAR-T cell culture. **(A)** Structures of the 4 CARs with the corresponding scFvs. **(B)** The binding domain of CEA in each scFv is shown. **(C)** Preparation and FCM detection schedule of CAR-T cells.

Four reported humanized scFvs (M5A, hMN-14, BW431/26, and C2-45) were synthesized (GenScript, China) and inserted into the pCDH lentiviral vector linked to the IgG4 hinge (G4h), CD28 transmembrane domain (28TM), CD28 intracellular signaling domains, 4-1BB intracellular signaling domains, and CD3 zeta ITAM domains. The EF1α promoter was used to drive CAR expression. The GFP sequence was also inserted downstream of each CAR-T sequence using the 2A protein (**Figure 2A**). Lentivirus was collected and purified from the supernatant of transfected HEK293T cells. To generate CAR-T cells, fresh primary human lymphocytes were obtained from healthy volunteer donors and cultured in GT-T561 (Takara) medium supplemented with 10% fetal bovine serum (FBS; Gibco). PBMCs were activated with immobilized GMP anti-CD3 (MACS, 170-076-116) and anti-CD28 (MACS, 170-076-117) antibodies. Then, the cells were transduced in 6-well plates (1×10^6^ cells per well, the multiplicity of infection of 3-5 per well) in the presence of polybrene. T cells were stimulated and expanded with IL-2 (100 U/ml) after viral transduction.

### Flow cytometric detection and analysis

We applied BD FACSCalibur and BD FACSAria II instruments for FCM detection. BD FACSDiva software and FlowJo software were used for analysis. FCM detection was performed according to the schedule shown in **Figure 2C**. CD3, CD4, CD8, and CAR expression were evaluated on days 6, 10, and 14, and memory T subsets were evaluated on days 10 and 14. We used His-tagged recombinant CEACAM5 (CEA-His; Sino Biological Inc., 11077-H08H-50) and biotin-protein L (GenScript, M00097) for specific detection of CAR expression. The following antibodies were used: anti-CD3-PE-Cy7 (BioLegend, 300420), anti-CD4-BUV395 (BD, 564724), anti-CD8-BV510 (BioLegend, 344732), anti-CD25-BV421 (BioLegend, 302630), anti-CD45RA-BV421 (BioLegend, 304130), anti-CD45RO-PerCP-Cy5.5 (BioLegend, 304222), AF-647-conjugated IgG fraction of mouse monoclonal anti-biotin (Jackson, 200-602-211) and anti-CD197-PE (BioLegend, 353204).

### CAR-T cell response to repeat CEA stimulation

Six-well plates were coated with the CEACAM5 protein (Human, Recombinant Fc Tag). On day 0, 2.5×10^6^ viable CAR-T cells were plated into the CEA-coated plates and cultured with 10% FBS medium in the absence of cytokines. The culture supernatant was collected on day 1 for measurement of secreted IFN-γ and IL-2 levels by ELISA. The expression of CD25 in CAR-T cells was measured after 48 hours of culture in CEA-coated plates. The medium was supplemented according to the growth of the cells, and the cells were counted every two days using AO/PI staining to evaluate cell proliferation. The proportion of CAR-positive cells was indicated by the rate of GFP-positive cells. The stimulation was repeated every 7 days until the GFP-positive CAR-T cells stopped expanding. The fold expansion of CAR-T cells was calculated, and after repeated stimulation, cumulative proliferation was analyzed.

### *In vitro* cytolytic assay

The cytolytic assay was conducted with an ACEA xCELLigence RTCA MP instrument and related protocols. Data were recorded based on cell attachment to the plate. In brief, tumor cells attached to the plate and induced an increase in the electrical index of the plate. When CAR-T cells were added and eliminated these attached tumor cells, the electrical index was altered and recorded. On the first day, 2-5 × 10^4^ tumor cells were added to each well of a 96-well plate. The electrical index was recorded every 15 minutes to monitor the attachment of tumor cells. Twenty-four hours later, CAR-T cells were added to each well at a certain E:T ratio. The electrical index was measured over the next 24 hours. Two replicates of each well were established. The specific cytolysis percentage was calculated based on the attenuation of the electrical index compared to the baseline index before the addition of CAR-T cells: % specific lysis = (baseline index - real-time index)/baseline index. Supernatants were collected 24 hours after the addition of CAR-T cells. These specimens were stored at -80°C and subjected to ELISA (IFN-γ, BD Biosciences, 4316955) with a related protocol.

### Mouse xenograft studies

At 6-8 weeks of age, female NOD.Cg-PrkdcscidIL2rgtm1Sug/JicCrl (NOG) mice (Vital River Laboratory Animal Technology Co., Ltd.; Beijing, China) were injected subcutaneously with 1×10^6^ (per mouse) LoVo cells and fed in a specific pathogen-free (SPF) environment at the animal facility of Southwest Hospital. The mice received humane care according to the criteria outlined in the “Guide for the Care and Use of Laboratory Animals” prepared by the National Academy of Sciences. Tumor volumes and tumor bioluminescence were confirmed before CAR-T cell infusion. After that, the mice were randomly and equally assigned to groups (n=4). Each mouse was injected with 1×10^7^ CAR-T cells *via* the tail vein 6 days after LoVo cell implantation. Tumor volumes were measured every 3-4 days and calculated with the following equation: volume= length × width × width/2. The mice were imaged weekly. During tumor volume measurement and bioluminescence detection, the evaluator was blinded to the group allocations. After the experiment, only living mice were included in the statistical analyses. Tumor volumes and tumor bioluminescence were compared among groups.

### Statistical analysis

Statistical analyses were performed with SPSS software (version 13.0) and GraphPad Prism software (version 8). Student’s t-test was used for comparisons between the two groups. ANOVA (randomized block design) was applied for comparisons among more than two groups. One-way ANOVA and Student’s t-test were applied for the comparison of tumor volumes among groups. The Shapiro−Wilk test was applied to confirm whether the data analyzed were normally distributed, and the F test was used to compare variances. In the figures, the data are presented as the means ± SDs or means ± SEMs, and the mean values were calculated from at least three independent experiments. The significance of differences was defined as follows: ns = not significant; * = p < 0.05; ** = p < 0.01; *** = p < 0.001.

## Results

### scFvs derived from M5A and hMN-14 showed higher affinity

We obtained and purified the scFv of each mAb with a purity of greater than 95%. The affinity of each scFv was measured with a gradient of 5 concentrations, and the K_D_ value was calculated (**Figure 1**). M5A (15.8 nM, **Figure 1A**) and hMN-14 (4.6 nM, **Figure 1B**) showed higher affinity for CEA than BW431/26 (229.2 nM, **Figure 1C**) and C2-45 (46.3 nM, **Figure 1D**).

### Generation and detection of CAR-T cells

Four scFvs derived from each mAb were generated and inserted into a 3^rd^-generation CAR backbone (**Figure 2A**). The CEA binding site for each mAb is shown (**Figure 2B**). The binding site of C2-45 was not identified, while the other three mAbs bound to the A3 domain of CEA (38, 39). The procedures for cell preparation were formulated, and FCM detection was performed on days 6, 10, and 14 (**Figure 2C**).

### M5A and hMN-14 CAR showed more stable and sustained binding to the CEA protein

**Figure 3:**
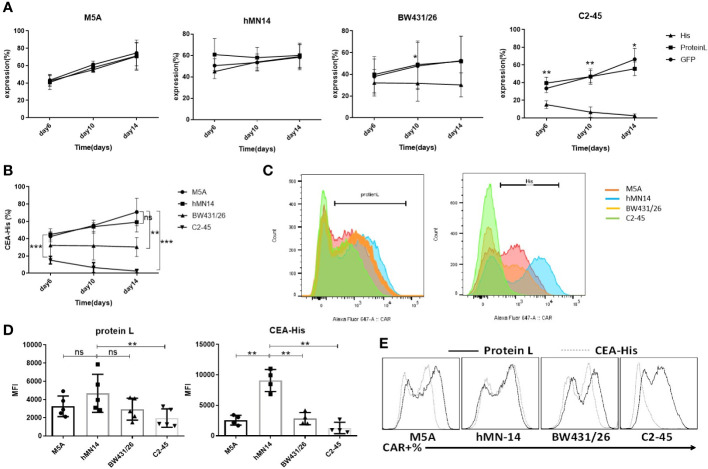
Comparison of the detection and expression of several scFvs CAR by flow cytometry. **(A)** The expression of the 4 CAR scFvs on the same cells was evaluated on days 6, 10, and 14 of cell culture using three methods: CEA-His, protein L and GFP. The values are presented as the mean ± SD of four independent experiments derived from 4 different donors (n=4). **(B)** When CAR expression was detected using CEA-His, the results for the 4 types of CAR-T cells were compared. hMN-14, BW431/26 and C2-45 CAR-T cells were compared to M5A cells. The values are presented as the mean ± SD of four independent experiments (n=4). **(C)** Representative FCM results for protein L and CEA-His in the 4 types of CAR-T cells. **(D)** The MFI of protein L and CEA-His was also measured and compared on day 10 of cell culture. The data are presented as the mean ± SD of five independent experiments (protein L) (n=5) and four independent experiments (CEA-His) (n=4). **(E)** HEK293T cells were transduced with each CAR viral vector, and CAR expression was measured by assessment of protein L and CEA-His. Representative FCM results from three independent experiments are shown (n=3). Statistical analysis was performed by a paired t-test. * = p < 0.05; ** = p < 0.01; and ns, not significant.

CAR expression was evaluated according to the schedule on days 6, 10, and 14. The expression of all CARs was stably detected with protein L and GFP at every time point (**Figure 3A**). When CEA-His was used to determine the proportion of CAR-positive cells, M5A and hMN-14 maintained stable detectability (**Figures 3A, B**). However, for BW431/26 CAR, the proportion of CAR-positive cells detected by CEA-His was significantly different from that detected by GFP and PL, especially on days 10 and 14. In addition, C2-45 was barely detected by CEA-His on days 10 and 14 (**Figures 3A, B**). The results showed that the binding of CEA-His to the BW431/26 and C2-45 CARs was significantly lower than that to the M5A and hMN-14 CARs, and showed decreasing trend with the extension of culture time (**Figure 3B**). We speculated that the difference in the original affinity of scFv for CEA was a possible reason for the difference in CAR binding. Moreover, the different influences of the antigen-recognition domain on each scFv changed the binding ability. The results were replicated in multiple donors (**Figure 3C**). The mean fluorescence intensity (MFI) was also measured and compared on day 10 of cell culture, and the MFI of the hMN-14 CAR, especially that bound to CEA-His, remained stronger (**Figure 3D**). The strongest affinity was one of the main contributions. To exclude T-cell-related expression bias, we separately transduced HEK293T cells with a lentivirus expressing each CAR. CAR expression was similar to that in T cells (**Figure 3E**).

### M5A CAR-T cells displayed higher proliferation but a more differentiated phenotype than other CAR-T cells

**Figure 4:**
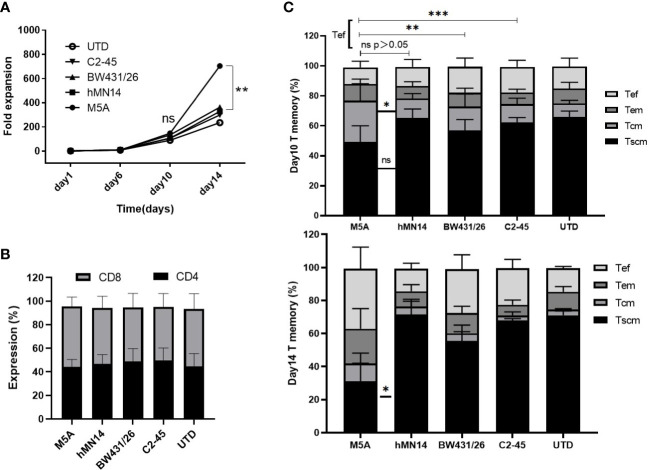
The proliferation and T-cell subsets of several types of CAR-T cells during culture. **(A)** Several types of CAR-T cells were cultured *in vitro* for two weeks and counted every four days by AO/PI staining. The fold expansion was calculated to evaluate the proliferative capacity. **(B)** The CD4-positive and CD8-positive populations in cultured cells were measured and compared. **(C)** The expression of CD45RA, CD45RO, and CCR7 was measured to determine the differentiation state of memory T-cell subsets by flow cytometry on day 10 and day 14: Tscm (CD45RA+CD197+), Tcm (CD45RA-CD45RO+CD197+), Tem (CD45RA-CD45RO+CD197-), and Tef (CD45RA+CD197-). All data were obtained from five independent experiments derived from 5 different donors (n=5). Statistical analysis was performed with a paired t-test in **(A, C)** and for **(B)** by one-way ANOVA in **(B)**. * = p < 0.05; ** = p < 0.01; *** = p < 0.001; and ns, not significant.

To determine whether different scFv CARs affect the cellular status, including the proliferative capacity and cell phenotype, the cell numbers were determined every four days, and the cell but M5A CAR-T cells displayed greater proliferation than the other CAR-T cells on day 14 (**Figure 4A**). However, the percentages of CD4 to CD8 were not different (**Figure 4B**). Memory T subsets were evaluated on days 10 and 14. M5A CAR-T cells contained more T_cm_ and fewer T_ef_ cells on day 10 of *in vitro* culture, while the T_ef_ population was increased on day 14 (**Figure 4C**). Based on the differences in cell proliferation and memory T-cell subsets, we confirmed that the M5A CAR induced enhanced promoting effects on cell proliferation and differentiation of memory T cells into effector cells at later culture time points, and the proliferation advantage was related to CAR rather than untransduced T cells.

### M5A CAR-T cells were less exhausted and secreted more cytokines in the CEA repeated stimulation assay

**Figure 5:**
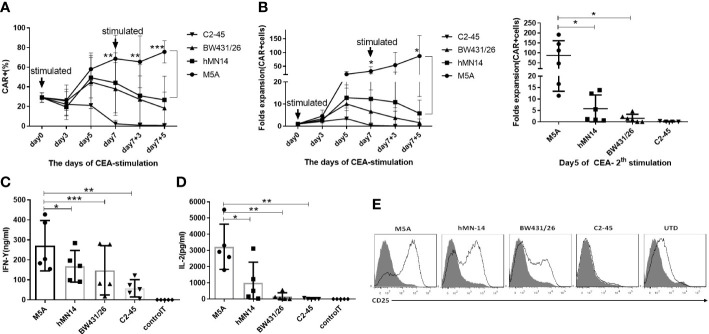
Evaluation of the CAR-T cell response to repeated CEA stimulation. All groups were compared with M5A (paired t-test). **(A)** Several types of CAR-T cells were incubated in 6-well plates coated with the CEACAM5 protein every seven days (1st and 2nd stimulation), and the percentage of CAR-positive cells was determined every 2-3 days by monitoring GFP expression. The values are presented as the mean ± SD of six independent experiments derived from 6 different donors (n=6) from different donors. **(B)** After two rounds of stimulation with CEA in the absence of cytokines, CAR-positive cells were counted, and the fold expansion was calculated by assessment of CAR-positive cells. The values are presented as the mean ± SD of six independent experiments (n=6) with technical duplicates. **(C, D)** The culture supernatant was collected 24 hours after CEA stimulation to measure the levels of secreted IFN-γ and IL-2 by ELISA. The values are presented as the mean ± SD of five independent experiments (n=5). **(E)** The CD25 expression of several scFvs CAR-T cells was detected 48 hours after CEA stimulation. Representative FCM results from three independent experiments are shown (n=3). Statistical analysis was performed by paired t-test. * = p < 0.05, ** = p < 0.01, *** = p < 0.001 and ns, not significant.

Because minor differences were observed among all 4 CAR-T cell phenotypes after cell culture, we performed repeated antigen stimulation assays to compare proliferation and cytokine secretion. The 4 CAR-T cell lines were repeatedly stimulated with CEA coated on the culture plate every 7 days. T cells were counted, and CAR expression was assayed every 2-3 days. Seven days after the first stimulation, the proportion of CAR-positive T cells among M5A CAR-T cells was significantly greater than that among the other CAR-T cells (**Figure 5A**). After the second stimulation, the population of CAR-T cells increased only among M5A CAR-T cells and gradually decreased in the other CAR-T cell populations (**Figure 5B**), M5A activated stronger intracellular signals to induce CAR-T cell responses. In addition, M5A CAR-T cells exhibited significantly greater cytokine secretion (IFN-γ and IL-2) than the other CAR-T cells (**Figures 5C, D**), that could also triggered M5A to gain more cell expansion. After the first stimulation, the phenotypic analysis revealed significantly upregulated expression of CD25 in M5A CAR-T cells. In contrast, C2-45 CAR-T cells barely responded to antigen stimulation, and CD25 was barely expressed (**Figure 5E**).

### CARs with scFvs with different affinities discriminated between cells expressing different levels of CEA

First, we performed cytolytic assays to compare the tumor elimination properties of the 4 types of CAR-T cells in a tumor cell line with high CEA expression (**Supplementary Figure S1**). CAR-T cells were cocultured with high-CEA-expression LoVo cells at effector:-target (E:T) ratios of 1:1 and 1:2 for 48 hours. All 3 tested CAR-T cells lysed LoVo cells in a concentration- and time-dependent manner. M5A and hMN-14 CAR-T cells showed better specific lysis ability and higher IFN-γ levels than the other 2 types of CAR-T cells. hMN-14 CAR-T cells exhibited the highest level of IL-2 secretion (**Supplementary Figure S1**). C2-45 CAR cells exhibited weaker tumoricidal capacity and little cytokine secretion. Considering the weak effects of C2-45, we did not use the C2-45 CAR in further experiments or comparisons.

**Figure 6:**
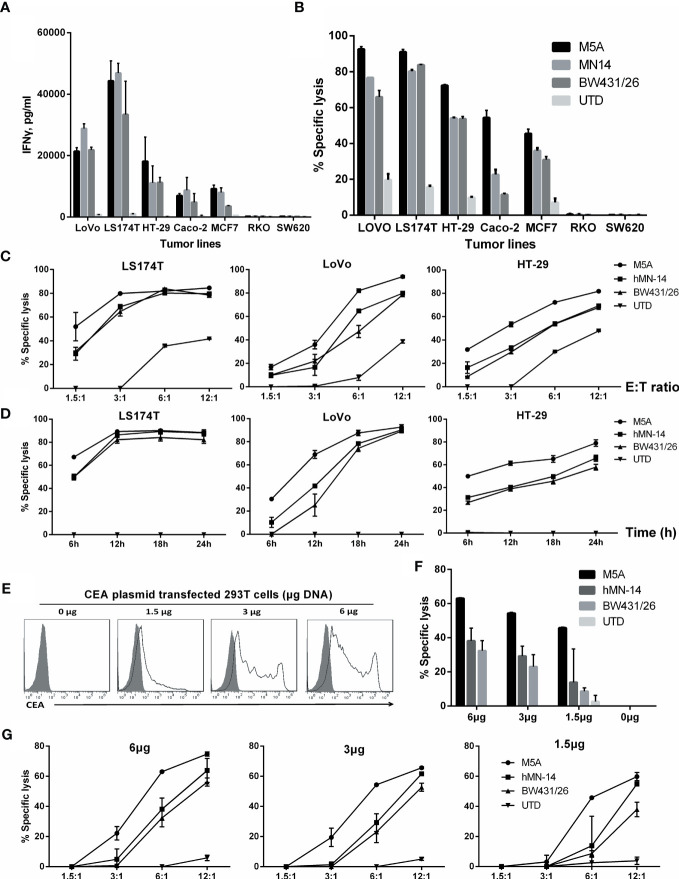
Comparison of the specific cytolytic activity of several types of CAR-T cells based on time and concentration. **(A)** IFN-γ was measured by ELISA in culture supernatants collected after 24 hours of coculture. **(B)** Specific cytolytic activity of CAR-T cells. Several types of CAR-T cells were incubated with tumor cell lines with different CEA expression levels for 8 hours at an E:T ratio of 6:1. Cytolysis at different E:T ratios after 8 hours **(C)** and at an E:T ratio of 3:1 after different durations **(D)** is shown. **(E)** HEK293T cells were transduced with different concentrations of a DNA plasmid expressing CEA. Membrane CEA expression was detected. **(F, G)** Specific cytolysis of each HEK293T cell line was measured. The other two groups were compared with M5A (ANOVA, randomized block design).

To further discriminate the function of these CAR-T cells, we evaluated several tumor cell lines with different levels of CEA expression. First, the expression of CEA in tumor cell lines was measured (**Supplementary Figure S2**). Then, after 24 hours of coculture of CAR-T cells with tumor cells, IFN-γ secretion was measured by ELISA. IFN-γ secretion was significantly higher in cell lines with high CEA expression (LoVo and LS174T) (**Figure 6A**). In cell lines with moderate or low CEA expression (HT-29, Caco-2, and MCF7), IFN-γ secretion was significantly lower, but the M5A and hMN-14 CAR-T cells showed a slight advantage. In CEA-negative cell lines (RKO and SW620), none of the 3 CAR-T cells exhibited obvious effects on cytokine secretion.

Next, we used a real-time, quantitative cell analysis system (xCELLigence RTCA System, Agilent) to determine the cytolytic activity of CAR-T cells against tumor cells with different levels of CEA expression. After 8 hours of coculture at an E:T ratio of 6:1, M5A, hMN-14, and BW431/26 CAR-T cells showed diverse cytolytic activity against tumor cell lines with different levels of CEA expression: high (LoVo and LS174T) and moderate or low (HT-29, Caco-2, and MCF7) (**Figure 6B**). M5A, hMN-14 and BW431/26 CAR-T cells showed high cytolytic activity against cells with high CEA expression, and M5A showed a slight advantage. The cytolytic activity of CAR-T cells against cell lines with moderate or low CEA expression gradually decreased, but M5A showed more stable cytolytic activity (**Figure 6B**). The preferential cytolytic activity of M5A was also confirmed in cytolytic assays with different E:T ratios and incubation times (**Figures 6C, D**). To further study the responses of several CAR-T scFvs to different CEA expression levels, we conducted cytolytic assays targeting HEK293T cells with exogenous expression of CEA. HEK293T cells were transfected with increasing concentrations of the CEA expression plasmid, and CEA expression was measured (**Figure 6E**). M5A also showed superior cytolytic activity against both high- and low-CEA-expressing HEK293T cells (**Figure 6F**). In addition, the cytolytic activity of M5A was confirmed at different E:T ratios (**Figure 6G**). The results showed that the cytolytic effect of CAR-T cells was significantly enhanced with increasing CEA expression on the HEK293T cell surface.

### M5A CAR-T cells had greater antitumor activity *in vivo*

**Figure 7:**
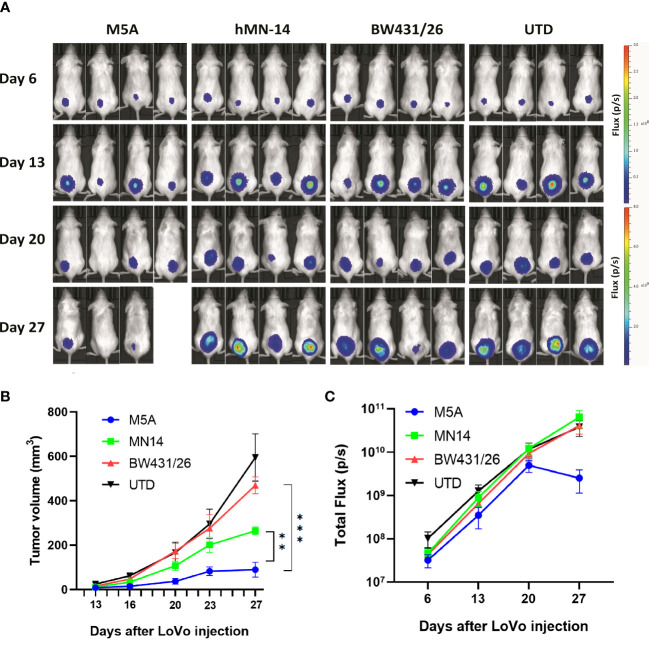
M5A CAR-T cells exhibited superior tumor suppression in the xenograft model in NOG mice. **(A)** Each mouse was implanted with 1× 10^6^ LoVo cells (Luc^+^) on day 1 and injected i.v. with 1 × 10^7^ CAR-T cells on day 7. Mice were imaged weekly. **(B)** Tumor growth was assessed by calculating the tumor volume. The values are presented as the means ± SEMs. The growth of tumors treated with M5A CAR-T cells was potently controlled compared with that of tumors in the other groups. **(C)** The total bioluminescence values were also recorded and compared. The values are presented as the means ± SEMs. Statistical analysis was performed by one-way ANOVA. * = p < 0.05; ** = p < 0.01; and ns, not significant.

We evaluated the antitumor activity of M5A, hMN-14, and BW431/26 CAR-T cells *in vivo* in a LoVo cell xenograft model. NOG mice were injected i.v. with 1 × 10^6^ LoVo cells genetically modified to express firefly luciferase. Six days later, the mice were injected intravenously (i.v.) with 1 × 10^7^ CAR-T cells without any preconditioning treatment. Control mice were injected with 1 × 10^7^ T cells with no CAR. The tumor burden was monitored by serial bioluminescence imaging every 7 days (**Figure 7A**). M5A CAR-T cells showed superior tumor suppression compared with that of the other CAR-T cells (**Figure 7B**). M5A CAR-T cells consistently exhibited preferential tumor suppression and were superior to BW431/26 and UTD (p < 0.001). According to the actual measurements on day 27, the tumor volume increased in the following order: M5A<hMN-14<BW431/26<UTD (**Figure 7B**). These results indicated optimal tumor suppression by M5A CAR-T cells. The total bioluminescence flux is shown in **Figure 7C**, and the values indicate a decreasing trend only in the M5A group and no significant difference in the other groups.

## Discussion

CEA is highly expressed in various types of solid tumors, especially some gastrointestinal tumors. Its high expression in tumors and detectability in serum make it a valuable tumor biomarker for clinical diagnosis. CEA expression is undetectable in most normal tissues, except for the surface epithelium of the tongue, the tracheal mucosa, and specific locations in the gastrointestinal tract, where it is expressed at low levels (23). The reactivity of CAR-T cells toward antigen-expressing cells is affected by the avidity of the CAR, which is based on its affinity and surface expression level. Thus, screening for antibodies with appropriate affinity is very important for the application of CEA-targeting CAR-T cells. BW431/26 is a humanized antibody with moderate affinity for CEA. We enrolled ten CRC patients for CAR-T therapy targeting CEA (NCT02349724) using the BW431/26 antibody as the source of scFv. No off-target or on-target/off-tumor side effects were observed. However, patients achieved only transient disease control, and none achieved remission. During CAR-T cell production, we observed unstable CAR, leading to some canceled infusions due to low CAR-positive rates. We suspected that this CAR instability attenuated the expected outcomes. Therefore, the development of better CAR-T cells with more stable expression and greater antitumor effects will improve clinical outcomes.

CEA-specific antibodies, such as T84.66, hMN-14, C2-45, BW431/26, MFE-23, H10 and F023C5, have been developed (38, 40–45). By analyzing the antigen recognition sites and clinical application specificity, we identified three more antibodies from previously reported sources. M5A, hMN-14, and BW431/26 are humanized mAbs and C2-45 is a fully human mAb derived from KM mice, and these mAbs are less likely than others to induce an immune response. The affinity of the scFv derived from these antibodies was measured using a protein expression system in *Escherichia coli*. All four scFvs bound to the CEA protein with their corresponding affinities, as previously reported. When we confirmed the CEA binding capacity after these scFvs were transduced into T cells, the C2-45-derived scFv showed a very low CEA protein-binding capacity. By increasing the amounts of CEA-his to at least 4 times the recommended dose, the results for BW431/26 and C2-45 showed only small increase, which were still far from the percentage of GFP. Previous studies have also reported that the antigen recognition properties of the scFv incorporated into a CAR can differ from those of the original antibody (46). scFvs expressed in mammalian cells undergo various posttranslational modifications, such as glycosylation. C2-45 might have undergone an unexpected modification and formed an unexpected structure different from that produced in *E. coli*. In addition, CAR aggregation can cause loss of antigen recognition properties, as well as increased differentiation of CAR-T cells (35). When we compared the Tscm and Tcm populations in C2-45 CAR-T cells with those in untransfected control T cells, there was no difference between the two groups. Therefore, the loss of C2-45 affinity is unlikely to be due to aggregation. The three other scFv-derived CARs retained their CEA binding capacity, M5A and hMN-14 were better than BW431/26.

In contrast to BW431/26, the expression of both M5A and hMN-14 was highly stable. There was no significant difference between M5A and hMN-14 in CAR expression. The structure and posttranslational modification of the scFv are predicted to affect the folding of the related CAR protein, leading to structurally unstable CARs that are degraded intracellularly or aggregated on the T-cell membrane. In the structures of BW431/26, M5A, and hMN-14, the complementarity-determining regions (CDRs) of M5A and hMN-14 were assembled in the same human antibody framework region (FR) and were different from those of BW431/26. The FR has been reported to affect the stability of the scFv structure, CAR expression, and KDR binding (47). Changing the FR of BW431/26 might improve CAR expression and stability.

The affinity of a CAR for antigens has been reported to affect the efficacy of CAR-T cells both *in vivo* and *in vitro* (24). CARs containing scFvs with low affinity have shown superior safety and efficacy compared with those of CARs containing scFvs with high affinity (25). Increasing the affinity did not significantly enhance CAR-T cell function and even worsened the on-target/off-tumor effect (26). However, when comparing scFvs derived from different hybridomas and targeting different epitopes, high-affinity scFvs exhibited superior tumor eradication (27, 28). The benefits of increasing or decreasing scFv affinity are discrepant in different types of CAR-T (48). BW431/26 showed the lowest affinity (229.2 nM), hMN-14 showed the highest affinity (4.6 nM), and M5A showed moderate affinity (15.8 nM). hMN-14 CAR-T cells showed the highest cytokine expression and BW431/26 CAR-T cells showed the lowest cytokine expression *in vitro* when cocultured with target cells. *In vitro*, M5A and hMN-14 CAR-T cells showed comparable cytotoxic activity and slightly higher cytotoxicity than BW431/26 CAR-T cells when cocultured with target cells. However, in the SCID mouse xenograft model, M5A CAR-T cells showed the most potent antitumor effect. These results suggest that appropriate affinity improves CAR-T cell function based on different types of CAR-T.

In summary, we screened and compared four CEA-targeting antibody-derived CAR-T cells. M5A CAR-T cells showed stable CAR expression, moderate affinity, moderate cytokine secretion, and superior antitumor ability *in vivo* and *in vitro*. Further clinical trials will be performed to test the clinical efficacy of these cells.

## Data availability statement

The original contributions presented in the study are included in the article/**Supplementary Material**. Further inquiries can be directed to the corresponding authors.

## Ethics statement

The animal study was reviewed and approved by Third military medical university(army medical university).

## Author contributions

CQ and ZY designed the experiments. YH, XH, ZY, and WZ constructed the plasmid and packaged the virus. CZ and LW conducted the experiments. QZ, JS, MW, JC, YX, YQ, YL, and YO developed the methodologies. ZY, CZ, and LW analyzed the data and wrote the manuscript. All authors contributed to the article and approved the submitted version.
