# Corrigendum: Screening and characterization of the scFv for chimeric antigen receptor T cells targeting CEA-positive carcinoma

**DOI:** 10.3389/fimmu.2025.1548247

**Published:** 2025-01-31

**Authors:** Chengcheng Zhang, Linling Wang, Qianzhen Zhang, Junjie Shen, Xia Huang, Meiling Wang, Yi Huang, Jun Chen, Yanmin Xu, Wenxu Zhao, Yanan Qi, Yunyan Li, Yanjiao Ou, Zhi Yang, Cheng Qian

**Affiliations:** ^1^ Department of Hepatobiliary Surgery, Southwest Hospital, Army Medical University, Chongqing, China; ^2^ Chongqing Key Laboratory of Gene and Cell Therapy, Institute of Precision Medicine and Biotechnology, Chongqing Precision Biotech Co. Ltd., Chongqing, China

**Keywords:** chimeric antigen receptor T cells, carcinoembryonic antigen, single-chain fragment variable, affinity, cell therapy

In the published article, there was an error in **Figure 7A** as published. Among a large amount of pictures for selection, we found two graphs identical since they were not classified correctly. The corrected **Figure 7A** and its caption appear below.

**Figure 7:**
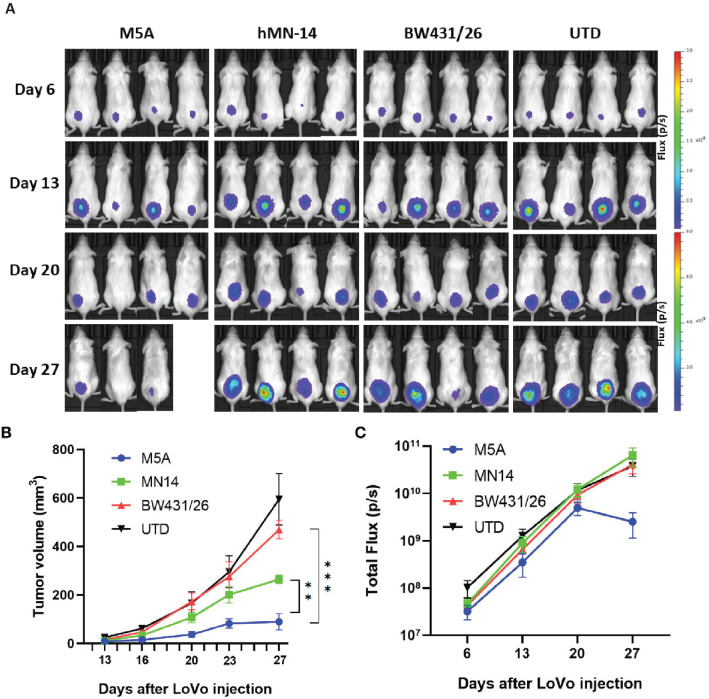
M5A CAR-T cells exhibited superior tumor suppression in the xenograft model in NOG mice. **(A)** Each mouse was implanted with 1× 10^6^ LoVo cells (Luc^+^) on day 1 and injected i.v. with 1 × 10^7^ CAR-T cells on day 7. Mice were imaged weekly. **(B)** Tumor growth was assessed by calculating the tumor volume. The values are presented as the means ± SEMs. The growth of tumors treated with M5A CAR-T cells was potently controlled compared with that of tumors in the other groups. **(C)** The total bioluminescence values were also recorded and compared. The values are presented as the means ± SEMs. Statistical analysis was performed by one-way ANOVA. * = p < 0.05; ** = p < 0.01; and ns, not significant.

The authors apologize for this error and state that this does not change the scientific conclusions of the article in any way. The original article has been updated.

