# Supplementary material for: Screening and characterization of the scFv for chimeric antigen receptor T cells targeting CEA-positive carcinoma
Source: Front Immunol. 2023 May 25;14:1182409. doi: 10.3389/fimmu.2023.1182409 (PMC10248079; doi:10.3389/fimmu.2023.1182409)
Supplement: Supplementary file 1 [file DataSheet_1.docx]

Supplementary Material

Screening and characterization of the optimal scFv for chimeric antigen receptor T (CAR-T) cells targeting CEA-positive carcinoma

**Chengcheng Zhang1†, Linling Wang2†**

*** Correspondence:**

Prof. Cheng Qian, Chongqing Precision Biotech Co., Ltd., Chongqing 400039, China.

Email: cqian8634@gmail.com

Dr. Zhi Yang， Chongqing Precision Biotech Co., Ltd., Chongqing 400039, China.

E-mail: [yz2003can@126.com](mailto:yz2003can@126.com)

^†^These authors contributed equally to this work.


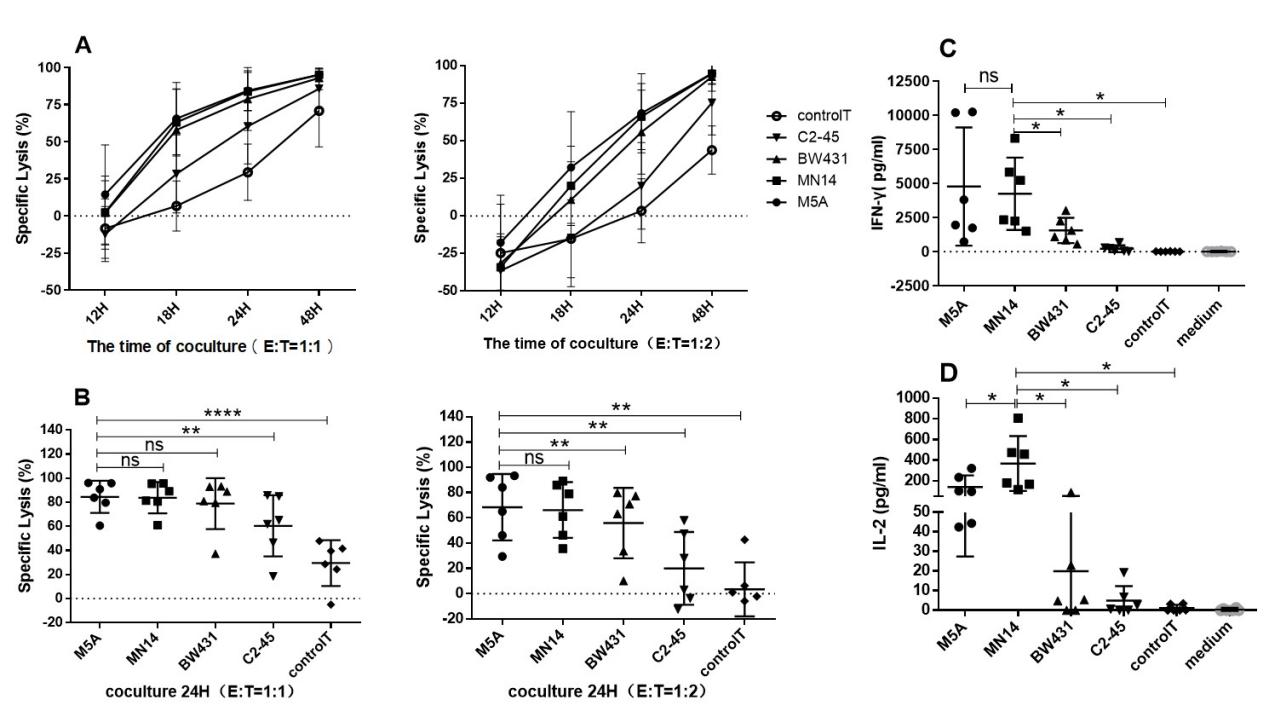


**Supplementary Figure 1.**The several scFv-CEA CART cells function of the specific lysis tumor cell line and the level of cytokine secretion. (A) The cells were co-cultured with CEA positive LoVo with effector-target ratio of 1:1 and 1:2 for 48 hours, in the different time calculated the specific lysis proportion. (B) CAR-T cells cocultured with LoVo with effector-target ratio of 1:1 for 24 hours, the specific lysis ability was compared. (C) The supernatant was collected after coculture 24 hours, effector-target ratio of 1:2, the levels of IFN-γ(C)and IL-2(D) release measured by ELISA. Values represent the average ± SD of duplicates of six independent experiments.

**Fig. S2**


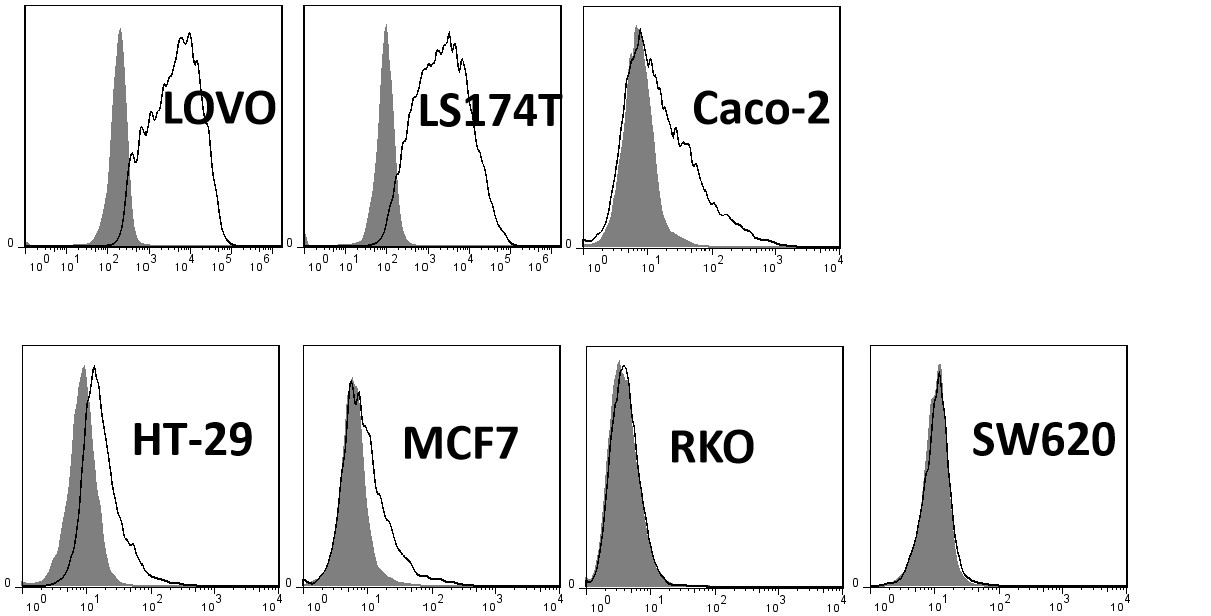


**Supplementary Figure 2.**The CEA expression in different CRC tumor cell lines.
